# miR-155 in the Resolution of Atherosclerosis

**DOI:** 10.3389/fphar.2019.00463

**Published:** 2019-05-14

**Authors:** Robyn Bruen, Stephen Fitzsimons, Orina Belton

**Affiliations:** Diabetes Complications Research Centre, School of Biomolecular and Biomedical Science, UCD Conway Institute, University College Dublin, Dublin, Ireland

**Keywords:** monocytes, macrophages, atherosclerosis, inflammation, miR-155

## Abstract

Atherosclerosis is a chronic progressive inflammatory disease where advanced lesions can eventually completely obstruct blood flow resulting in clinical events, such as a myocardial infarction or stroke. Monocytes and macrophages are the dominant biologically active immune cells involved in atherosclerosis disease and play a pivotal role during initiation, progression, and regression of disease. Altering macrophage inflammation is critical to induce regression of atherosclerosis and microRNAs (miRs) have emerged as key regulators of the macrophage phenotype. MiRs are small noncoding RNAs that regulate gene expression. They are dysregulated during atherosclerosis development and are key regulators of macrophage function and polarization. MiRs are short nucleotide transcripts that are very stable in circulation and thus have potential as therapeutics and/or biomarkers in the context of atherosclerosis. Of relevance to this review is that inhibition of macrophage-specific miR-155 may be a viable therapeutic strategy to decrease inflammation associated with atherosclerosis. However, further studies on these miRs and advancements in miR therapeutic delivery are required for these therapeutics to advance to the clinical setting. Conjugated linoleic acid (CLA), a pro-resolving lipid mediator, is an agonist of the peroxisome proliferator-activated receptor (PPAR)-γ. The biological activities of CLA have been documented to have anti-atherogenic effects in experimental models of atherosclerosis, inducing regression and impacting on monocyte and macrophage cells. Our work and that of others on PPAR-γ agonists and polyunsaturated fatty acids have shown that these mediators regulate candidate miRNAs and promote pro-resolving atherosclerotic plaque microenvironments.

## Atherosclerosis

Atherosclerosis is a chronic progressive disease that is characterized by accumulation and deposition of lipids and fibrous elements, coupled with an inflammatory response resulting in the development of lesions. Lesions develop in the tunica intima of large- and medium-sized arteries (Ross, 1993), following endothelial cell (EC) damage due to hyperlipidemia, hyperglycemia, hypertension, and inflammation (Knowles et al., 2000). The earliest clinical hallmark of a developing atherosclerotic lesion is the accumulation of lipid-laden macrophages termed foam cells which aggregate to form the “fatty streak” (Gerhard and Duell, 1999).

## Atherosclerosis and Inflammation

Inflammation plays a pivotal role in atherosclerosis disease progression. Modified lipids such as oxidized low-density lipoprotein (oxLDL) cholesterol stimulate ECs to secrete pro-inflammatory mediators including cytokines and adhesion molecules which facilitate monocyte adhesion and subsequent migration, resulting in macrophage differentiation and expansion.

Monocytes originate from innate precursor myeloid cells in the bone marrow (Thomas et al., 2017). They account for ~10% of all leukocytes and circulate in the blood for 1–3 days (Ziegler-Heitbrock et al., 1993; Ley et al., 2011). Monocytes repopulate macrophage populations, control homeostasis, and participate in inflammatory responses switching on both innate and adaptive immune responses (Gerhard and Duell, 1999; Swirski et al., 2007). Monocytes are a heterogeneous cell population and different subsets are associated with changes in inflammation status (Ley et al., 2011). Murine monocytes are identified by surface expression of CD115, CD11b, F4/80, and chemokine receptors CCR2 and CX3CR1 (Patel and Zhang, 2017), which identify two distinct monocyte populations in mice, inflammatory and patrolling. The three main monocyte (Mo) subsets in humans are described as Mo1 classical, Mo2 intermediate and Mo3 nonclassical or patrolling monocytes.

**Figure 1:**
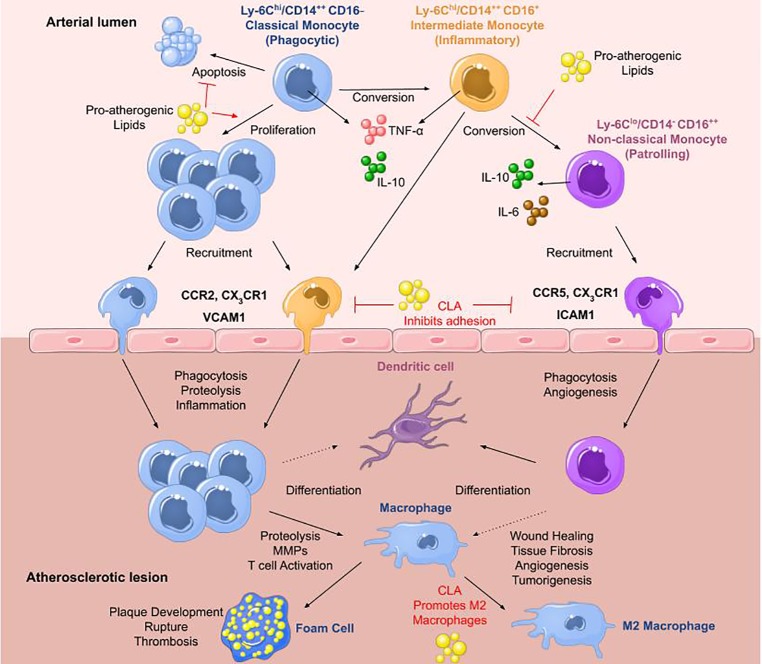
Monocytes in atherosclerosis. The three main monocyte subtypes are Mo1, Mo2, and Mo3. Mo1 are classical monocytes defined as inflammatory, phagocytic, CD14^++^ CD16^−^ in humans and Ly6C^hi^ in mice. They infiltrate lesions through CCR2 and differentiate into macrophages that can readily transform into foam cells. They secrete IL-10 and high levels of TNF-α upon stimulation with a toll-like receptor 4 agonist. Mo2 monocytes are termed intermediate monocytes and only found in humans defined by CD14^++^ CD16^+^. They are most similar to murine Ly6C^hi^ inflammatory monocytes. They can convert into Mo3 nonclassical anti-inflammatory monocytes differentiating into dendritic cells or pro-inflammatory macrophages most likely contributing to disease progression. Mo3 patrolling monocytes are identified as CD14^−^ CD16^++^ in humans and Ly6C^lo^ in mice. They secrete high levels of IL-10 and IL-6 with roles in wound healing and angiogenesis. They infiltrate plaques through CCR5. In atherosclerosis, high levels of pro-atherogenic lipids promote proliferation of Mo1 monocytes, inhibiting their apoptosis, and block the conversion of Mo2 intermediate monocytes into Mo3 nonclassical monocytes. Pro-resolving lipid mediators such as CLA can inhibit monocyte adhesion to ECs and promote M2 macrophage differentiation (adapted from Ley et al., 2011).

Classical or pro-inflammatory Mo1 monocytes represent 80–90% of the monocyte population and are typically described as CD14^++^ CD16^−^ in humans (Ley et al., 2011) or Ly6C^+^ in mice (Swirski et al., 2007). Upon stimulation, they secrete high levels of interleukin (IL)-10 (Swirski et al., 2007), and activation with toll-like receptor 4 agonists results in the secretion of tumor necrosis factor (TNF)-α, IL-6, and IL-1β (Ley et al., 2011; Italiani and Boraschi, 2014), whereas stimulation with a toll-like receptor 3 agonist results in interferon (IFN)-α secretion. They extravasate into the blood in a CCR2-MCP-1-dependent manner where they mediate inflammatory responses (Boyette et al., 2017). Mo2 intermediate monocytes are only found in humans characterized by CD14^++^ CD16^+^ and are most similar to human classical and murine Ly6C^+^ monocytes (Ley et al., 2011; Boyette et al., 2017; Patel and Zhang, 2017). They secrete high levels of TNF-α and low levels of anti-inflammatory IL-10 (Italiani and Boraschi, 2014) and are increased in patients with arterial disease compared to healthy controls (Tsujioka et al., 2009). It has been shown that days following myocardial infarction, there is an increase in the classical Mo1 monocyte population, whereas days later, intermediate Mo2 monocytes prevail (Tsujioka et al., 2009). Nonclassical Mo3 monocytes are identified by CD14^−/+/lo^ CD16^++^ in humans (Ley et al., 2011) or Ly6C^−^ in mice and are patrolling or resident monocytes (Ziegler-Heitbrock et al., 1993; Patel and Zhang, 2017). Upon stimulation, they secrete lower levels of TNF-α, IL-6, and IL-1β, and higher levels of IL-10 compared to other subsets (Ley et al., 2011) and they expand under conditions of stress (Patel and Zhang, 2017). Mo3 monocytes migrate to sites of damaged vasculature to promote wound healing (Figure 1).

Macrophage contribution to plaque development was identified when the macrophage-colony stimulating factor (M-CSF)-deficient osteopetrotic apolipoprotein E knockout (ApoE^−/−^) mouse was shown to have an 86% decrease in lesion volume (Moore and Tabas, 2011). M-CSF drives the differentiation of monocytes into unpolarized M0 macrophages *in vitro* (Mosser and Edwards, 2008). M1 “classical” macrophages are pro-inflammatory, secreting the pro-inflammatory cytokines IL-1β, IL-6, IL-12, and TNF-α and are also characterized by increased expression of inducible nitric oxide synthase (iNOS), cyclooxygenase-2, and the generation of reactive oxygen species (Butcher and Galkina, 2011). The effects of macrophage-derived pro-inflammatory cytokines on vascular cells is well documented, where they contribute to EC dysfunction, reducing EC secretion of endothelial nitric oxide synthase and driving oxidative stress. M1 macrophages have been implicated in the formation of the necrotic core, plaque destabilization, and thrombus formation due to their ability to phagocytose oxLDL and secrete matrix metalloproteinase (MMP)-1, MMP-3, and MMP-9 (Boyle et al., 2011). M2 “alternative” macrophages were first derived from monocytes using M-CSF and IL-4 (Gordon and Martinez, 2010) and are characterized by expression of CD206. More recently, M2 subsets such as M2a, M2b, and M2c macrophages have been identified, where M2a macrophages are derived from IL-4 and IL-13, M2b macrophages from IL-1β or lipopolysaccharide (LPS), and M2c macrophages from IL-10, transforming growth factor β or glucocorticoids (Wolfs et al., 2011). In atherosclerotic plaques, M2 macrophages promote wound healing, matrix remodeling, efferocytosis, and fibroblast recruitment (Butcher and Galkina, 2011; Huang et al., 2012) and are localized far from the lipid core, in contrast to M1 macrophages. M2 macrophages are unable to efficiently phagocytose oxLDL but are professional efferocytes with the ability to promote secretion of MMP-11 and MMP-12 (Boyle et al., 2011; Huang et al., 2012). This suggests that M2 macrophages mediate pro-resolving roles in the clearance of apoptotic cells in early atherosclerosis but may play a role in plaque destabilization in later stages of disease.

## Conjugated Linoleic Acid and Atherosclerosis

Conjugated linoleic acid (CLA) is a generic term denoting a group of naturally occurring isomers of linoleic acid (18:2, n6), that differ in the position or geometry [i.e., cis (c) or trans (t)] of their double bonds (Eder and Ringseis, 2010). There are 28 CLA isomers with c9,t11-CLA, which accounts for ~80% of CLA intake in the diet and t10,c12-CLA is the most abundant. The biological activities of CLA have been documented to have anti-atherogenic effects in an experimental model of atherosclerosis when administered in an 80:20 blend of its two most abundant isomers c9,t11-CLA and t10,c12-CLA, respectively (Toomey et al., 2006).

Our previous work, coincident with that of others, has shown that the CLA 80:20 blend induces resolution of pre-established atherosclerosis in ApoE^−/−^ mice. In comparison with controls, CLA-fed mice also had decreased aortic macrophage accumulation, decreased CD36 expression (Toomey et al., 2006), increased aortic peroxisome proliferator-activated receptor (PPAR)-α and PPAR-γ expression, and negative regulation of pro-inflammatory gene expression, suggesting that CLA exerts its pro-resolving effects in part *via* activation of PPARs (McClelland et al., 2010; McCarthy et al., 2013a,b). In more recent studies, it was shown that CLA isomers in an 80:20 blend induce M2 macrophages (de Gaetano et al., 2015).

Furthermore, in the ApoE^−/−^ model of atherosclerosis, CLA promotes a pro-resolving microenvironment, and we have identified that the monocyte/macrophage is the cellular target through which CLA mediates its effect (Toomey et al., 2006). CLA also inhibits monocyte adhesion to ECs, monocyte migration to monocyte chemoattractant protein-1 (MCP-1), and decreases MCP-1 production in part *via* a PPAR-γ-dependent mechanism (McClelland et al., 2010). This implies CLA is a potent inhibitor of monocyte function and may play a role in regulating the migratory monocytes in atherosclerosis.

Monocyte differentiation into macrophage subsets is critical for either promoting development or inducing resolution of atherosclerosis. The M1 macrophage content of atherosclerotic plaques is associated with the clinical incidence of ischemic stroke and increased inflammation (Brown et al., 2002) and it has been shown that there is an M2 to M1 switch during plaque progression suggesting that interventional tools, able to revert the macrophage infiltrate toward the M2 phenotype, may exert an athero-protective action. CLA in an 80:20 blend of c9,t11:t10,c12-CLA impacts on macrophage polarization by reducing CD68 expression of M1 macrophages and increasing CD163 and CD206 expression associated with M2 macrophages, in human peripheral blood mononuclear cell (PBMC)-derived macrophages (de Gaetano et al., 2015). These findings have been confirmed *in vivo* where CLA supplementation in ApoE^−/−^ mice induced the anti-inflammatory M2 phenotype *via* increasing IL-10 production in atherosclerosis regression (McCarthy et al., 2013a,b). This suggests that CLA primes the monocyte/macrophage toward a pro-resolving M2 phenotype to exert athero-protective effects. Further understanding of the pathways through which CLA mediates its effect on monocytes and macrophages is critical in identifying regulators that drive atherosclerotic regression including microRNAs (miRs) which govern macrophage phenotype. The effects of CLA on miRs have been previously documented in adipose tissue (Parra et al., 2010; Qi et al., 2015), intestinal epithelial cells (Daimiel-Ruiz et al., 2015), and ovarian cancer cells (Shahzad et al., 2018).

## miR Function

miRs are short noncoding RNAs, approximately 20 nucleotides in length. In a *Caenorhabditis elegans* model, it was demonstrated that *lin-4* transcripts, approximately 22 and 61 nucleotides in length, did not encode for a protein but regulated the messenger RNA (mRNA) of *lin-14* by inhibiting protein translation (Lee et al., 1993). miRs regulate gene expression through inhibition of translation (Bartel, 2004). Through binding with an RNA-induced silencing complex, the nucleotide sequence of the miR allows targeted base pairing with the 3′ untranslated regions of complementary mRNA (Hammond et al., 2000; Martinez et al., 2002). MiR sequence-specific silencing of mRNA can occur *via* two mechanisms: enzymatic cleavage of the transcript occurs if there is sufficient complementarity of the miR-mRNA sequences, or *via* translational repression if there is a lack of complementarity yet still some complementary miR sites present on the mRNA (Zeng et al., 2002, 2003). A single miR can target multiple transcripts and a single gene can be under the control of multiple miRs.

## Anti-Inflammatory Dietary Compounds and miRs

Although the effects of CLA on miRs in the context of atherosclerosis remain to be elucidated, in the context of myocardial infarction, treatment of mice with CLA in conjunction with nitrite improved heart function and induced miR-499 (Qipshidze-Kelm et al., 2013). Other anti-inflammatory dietary compounds function in part by downregulation of miR-155, a key regulator of inflammation, these include resveratrol (Tili et al., 2010), curcumin (Ma et al., 2017), apigenin (Arango et al., 2015), and quercetin (Boesch-Saadatmandi et al., 2011). The polyunsaturated fatty acids, docosahexaenoic acid and arachidonic acid, significantly decrease miR-155 in murine macrophages stimulated with LPS (Roessler et al., 2017). In addition, PPAR-γ agonists, rosiglitazone and telmisartan, decrease miR-155 in pre-adipocytes and in adipose tissue (Li et al., 2015; Peshdary and Atlas, 2018). Interestingly, CLA, a PPAR-γ agonist, increases aortic IL-10 secretion and increases phosphorylated signal transducer and activator of transcription (STAT)-3 signaling in the resolution of atherosclerosis (McCarthy et al., 2013a,b). It has been documented that IL-10 inhibits the BIC gene which encodes for miR-155 *via* a STAT-3-dependent mechanism (McCoy et al., 2010). miR-155 is one of several miRs that regulates inflammation and may be of clinical significance for novel therapeutics or prognostic indices of atherosclerotic disease progression and regression.

## miRs in Atherosclerosis

Multiple reviews have highlighted miR dysregulation in atherosclerosis progression (Small et al., 2010; Santovito et al., 2012; Condorelli et al., 2014). Several miRs, including miR-155, are upregulated in human cardiac disease (Krishnan et al., 2017) and miR-155 is significantly increased in plasma and plaques in atherosclerotic patients (Li et al., 2016). Given miR stability in circulation and their ability to regulate gene expression, they have potential in diagnostics (Fichtlscherer et al., 2010), prognostics (Karakas et al., 2017), and therapeutics. miRs involved in governing macrophage polarization and the distinct miR profiles of the M1 and M2 macrophage have been reviewed previously (Essandoh et al., 2016; Li et al., 2018) and miR-155 and miR-33 are emerging targets in macrophages to reduce atherosclerosis development. miR-33 inhibition in macrophages enhances cholesterol efflux (Najafi-Shoushtari et al., 2010) and reduces atherosclerotic lesions in mice (Horie et al., 2012; Ouimet et al., 2015). However, miR-155 may be a more *via*ble target given its role in determining macrophage phenotype.

**Figure 2:**
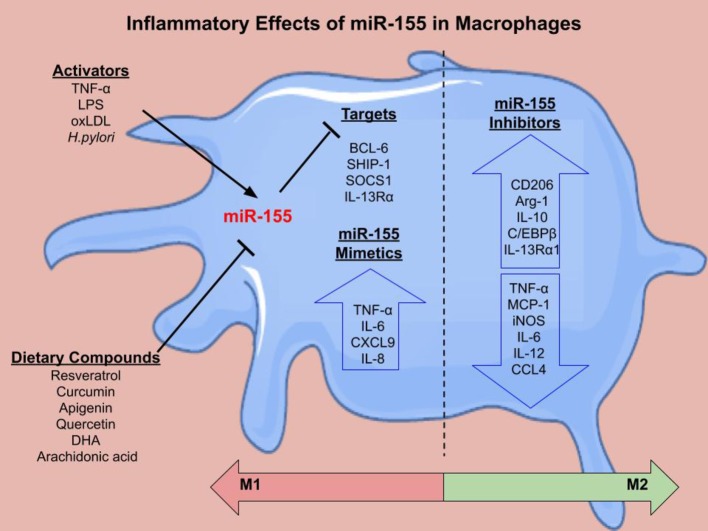
The inflammatory effects of miR-155 in macrophages. There are several activators of miR-155 including LPS, TNF-α, oxLDL, and *H.pylroi*. miR-155 can in turn inhibit gene expression. Several dietary compounds including the polyunsaturated fatty acids, docosahexaenoic acid (DHA) and arachidonic acid, can inhibit miR-155. Validated targets of miR-155 in macrophages include B Cell CLL/Lymphoma 6 (BCL-6) (Nazari-Jahantigh et al., 2012), inositol polyphosphate-5-phosphatase D (SHIP-1) (O’Connell et al., 2009), suppressor of cytokine signaling 1 (SOCS1) (Wang et al., 2010), and IL-13 receptor subunit α 1 (IL-13Rα1) (Martinez-Nunez et al., 2011). miR-155 mimetics cause increased pro-inflammatory cytokine secretion (TNF-α, IL-6, CXCL9, and IL-8). miR-155 inhibition decreased pro-inflammatory cytokines and chemokines and increased M2 markers. Overall, the literature suggests that miR-155 mimetics shift macrophages toward an M1 phenotype, while miR-155 inhibition skews the macrophage toward an M2 phenotype, however this can be context dependent.

miR-155 is encoded from the BIC gene in response to monocyte and macrophage stimulation. LPS upregulates miR-155 expression in THP-1 monocytes and macrophages (Taganov et al., 2006; Graff et al., 2012). Furthermore, miR-155 is increased during phorbol 12-myristate 13-acetate-stimulated differentiation of THP-1 monocytes to macrophages (Forrest et al., 2010). OxLDL also promotes miR-155 expression in PBMCs (Chen et al., 2009) and THP-1 macrophages (Li et al., 2016). In bone marrow-derived macrophages (BMDMs) from wild type (WT) mice, IFN-β and IFN-γ increased miR-155 expression *via* TNF-α autocrine signaling (O’Connell et al., 2007). Polarization of PBMC-derived macrophages (Graff et al., 2012), THP-1 cells, and BMDMs (Cai et al., 2012) to the M1 phenotype (induced by IFN-γ and LPS) also resulted in increased miR-155 expression. There are multiple targets of miR-155 in macrophages that regulate inflammation (Figure 2).

## miR-155 in Macrophages and Mouse Models of Atherosclerosis

miR-155 is upregulated in response to inflammatory stimuli where nuclear factor-кB directly binds to the promoter of pri-miR-155/BIC gene (Li et al., 2016). Several *in vitro* experiments have attempted to elucidate whether miR-155 increases or decreases monocyte/macrophage inflammation. One study showed that miR-155 is anti-inflammatory and mediates its effects through degradation of calcium-regulated heat stable protein 1 which is critical for the stabilization of TNF-α mRNA (Li et al., 2016). Transfection of miR-155 mimic into THP-1 macrophages followed by oxLDL stimulation decreased TNF-α and increased oxLDL uptake, while miR-155 inhibitors had opposing effects (Li et al., 2016).

However, several other studies report that miR-155 has pro-inflammatory effects. Transfection of M0 THP-1 macrophages with a miR-155 mimic resulted in increased IL-6, TNF-α, and CXCL9 (Graff et al., 2012). PBMCs from *Helicobacter pylori*-infected patients had increased miR-155 expression. Transfection of miR-155 mimics into these infected macrophages increased cytokine secretion of IL-10, TNF-α, and IL-8 (Yao et al., 2015).

miR-155 inhibition in BMDMs from WT mice demonstrated that miR-155 is critical in sustaining the pro-inflammatory response. Pro-inflammatory LDL receptor (LDLR)-related protein 1 antagonist activity was blocked by miR-155 inhibition, as measured by decreased expression of TNF-α, IL-6, and CCL4 when stimulated with LDLR-related protein 1 (Mantuano et al., 2016). TNF-α has been shown to increase miR-155 which in turn may function in a positive feedback loop that sustains inflammation. In a separate study, inhibition of miR-155 in WT macrophages increased transcription factor, CCAAT enhancer binding protein beta, and downstream arginase-1 expression which are associated with an M2 phenotype (Arranz et al., 2012). miR-155 inhibition also reduced LPS induction of iNOS and overexpression of miR-155 had directly opposing inflammatory effects (Arranz et al., 2012).

M2 macrophages are essential for regression of atherosclerosis. Inhibition of miR-155 in M1s using antisense oligonucleotides resulted in an increase in M2 markers arginase-1, chitinase 3-like 3, CD206, resistin-like molecule-α, and IL-10, and a decrease in M1 markers TNF-α, iNOS, and IL-12 (Cai et al., 2012). Interestingly, transfection of a miR-155 mimic into M2 macrophages skewed them toward an M1 macrophage profile. M0s treated with pre-miR-155 mimics prior to M2 polarization resulted in a suppressed M2 phenotype following stimulation with IL-4. Depleting miR-155 in M0s followed by stimulation with LPS and IFN-γ resulted in a decreased M1 phenotype (Cai et al., 2012). In addition, miR-155 inhibition in macrophages increased IL-13 receptor alpha subunit 1, which facilitated an IL-13-mediated increase in STAT-6 activation and upregulation of IL-13 regulated genes that are important in the development of the M2 phenotype (Martinez-Nunez et al., 2011). These studies demonstrate that miR-155 is critical in orchestrating the inflammatory response and may be a viable target to promote M2 polarization or to reprogram M1 macrophages to reduce chronic inflammation, which is essential for regression of atherosclerosis (Figure 2).

To date, studies on the effects of miR-155 deletion *in vivo* are conflicting. ApoE^−/−^ mice with a leukocyte-specific miR-155 deficiency had decreased plaque size and number of lesional macrophages following partial carotid ligation (Nazari-Jahantigh et al., 2012). Furthermore, macrophages derived from this model had lower levels of MCP-1 when activated. Similarly, ApoE^−/−^miR-155^−/−^ mice had decreased macrophage inflammation and reduced atherosclerotic lesion development. The regulatory effects of miR-155 on leukocyte cells were confirmed through transplantation of miR-155-deficient bone marrow into ApoE^−/−^ mice which also halted atherogenesis (Du et al., 2014). Other studies have demonstrated that injection of antagomir-155 attenuated atherosclerosis development and progression in ApoE^−/−^ mice (Yang et al., 2015; Ye et al., 2016). In contrast, Ldlr^−/−^ mice transplanted with miR-155-deficient bone marrow had increased atherosclerotic plaques, elevated levels of pro-inflammatory monocytes, and decreased IL-10 production from peritoneal macrophages (Donners et al., 2012). These contradictory results may be due to the different animal models used. After 3 months on a Western diet, ApoE^−/−^ mice have elevated plasma cholesterol, larger aortic root lesion with macrophage-dense necrotic cores, and increased smooth muscle cells in comparison to Ldlr^−/−^ mice (Roselaar et al., 1996). Therefore, the ApoE^−/−^ mouse model may be more reflective of advanced atherosclerosis, suggesting that miR-155 may have stage-specific effects during atherosclerotic lesion development.

## miR-155 Therapeutics in Clinical Trial and miR Delivery

While no miR therapeutics have entered clinical trial for the treatment of atherosclerosis, two miR-155 inhibitors are currently under development. MRG-106, a miR-155 inhibitor, administered intratumorally, was well tolerated, had on-target activity, and had promising preliminary results in a phase 1 clinical trial in 6 patients with the mycosis fungoides form of cutaneous T-cell lymphoma (Querfeld et al., 2016). MRG-106 is currently in the phase 2 SOLAR trial (NCT03713320). MRG-107 is another anti-miR-155 therapy currently under development for treatment of Amyloid Lateral Sclerosis. However, several miR therapeutic clinical trials have been discontinued, including studies on MRX34 (NCT 01829971), RG-125 (NCT02826525), and RG-012 (NCT02855268).

Delivery of the miR therapeutic to the desired site of action is critical to prevent off-target effects. Cheng et al. combined a peptide with a low pH-induced transmembrane structure (pHLIP) to a peptide nucleic acid anti-miR, specific for miR-155 (Cheng et al., 2015). This delivery vector, pHLIP-anti-miR-155, can only be transported through the plasma membrane under acidic conditions such as those located in solid tumors. In two mouse tumor models, intravenous administration of this construct inhibited miR-155, had no systematic toxicity, and reduced metastasis. RNA-sequencing and bioinformatic analysis showed that 25% of the genes upregulated by pHLIP-anti-miR-155 were associated with cell adhesion and leukocyte transendothelial migration (Cheng et al., 2015). Whether this technology could be effective in targeting atherosclerotic plaques directly remains to be investigated. pHLIP-anti-miR-155 functioned in solid tumors which have a pH of approximately 6. In contrast, plaque pH is heterogenous, lipid-rich regions have a pH 7.15 whereas calcified areas had a pH 7.73 (Naghavi et al., 2002). Young et al. used a “blockmir” technology oligonucleotide-based drug, CD5–2, to selectively inhibit the miR-27a binding site in vascular endothelial-cadherin (Young et al., 2013). Delivery of CD5–2 intravenously was shown to be effective in reducing vascular leak and inflammation in animal models of retinopathy (Ting et al., 2018). Further advancements in miR therapeutic delivery to the desired site of action may facilitate clinical development.

## Conclusion

Atherosclerosis is an inflammatory disease where monocyte/macrophage cells are the dominant biologically active immune cells. The athero-protective effects of CLA during an experimental *in vivo* model of atherosclerosis regression identified the monocyte/macrophage as the cellular target. Given the role of miRs in macrophage polarization, they are likely to be critical regulators in the regression of atherosclerosis. miR-155 is upregulated in response to pro-inflammatory stimuli and its inhibition may be a viable strategy to reduce the inflammatory response. However, given some conflicting results, more studies are required to investigate the stage-specific effects of miR-155 inhibition during atherosclerosis progression. However, further development of miRs in clinical trials and improved methods of miR delivery could lead to development of macrophage-specific miR therapeutics in the context of atherosclerosis.

## Author Contributions

RB and SF contributed equally to the preparation and writing of this manuscript, supervised by OB who edited the manuscript. All authors approved the final manuscript.

### Conflict of Interest Statement

The authors declare that the research was conducted in the absence of any commercial or financial relationships that could be construed as a potential conflict of interest.

## Abbreviations

ApoE^−/−^: Apolipoprotein E knockout
BMDM: Bone marrow-derived macrophage
c: cis
CLA: Conjugated linoleic acid
EC: Endothelial cell
IFN: Interferon
IL: Interleukin
iNOS: Inducible nitric oxide synthase
LDLR: LDL receptor
LPS: Lipopolysaccharide
MCP-1: Monocyte chemoattractant protein-1
M-CSF: Macrophage-colony stimulating factor
miR: microRNA
MMP: Matrix metalloproteinase
Mo: Monocyte subset
mRNA: Messenger RNA
oxLDL: Oxidized low-density lipoprotein
PBMC: Peripheral blood mononuclear cell
pHLIP: pH-induced transmembrane structure
PPAR: Peroxisome proliferator-activated receptor
STAT: Singal transducer and activator of transcription
t: trans
TNF: Tumor necrosis factor
WT: Wild type.
